# Painful Unilateral Knee Snapping after Hyperextension Injury and Meniscus Tear

**DOI:** 10.1055/s-0043-1777329

**Published:** 2023-12-06

**Authors:** Phillip Karsen, Joseph Brinkman, Jonathan Day, Daniel McGurren, Karan Patel

**Affiliations:** 1 Division of Sports Medicine, Department of Orthopedic Surgery, Mayo Clinic College of Medicine and Science, Mayo Clinic, Phoenix, Arizona; 2 Department of Orthopedic Surgery, Mayo Clinic, Phoenix, Arizona; 3 Department of Orthopedic Surgery, Georgetown University, Washington, DC; 4 Department of Physical Therapy, Mayo Clinic College of Medicine and Science, Mayo Clinic, Phoenix, Arizona; 5 Department of Orthopedics, Mayo Clinic College of Medicine and Science, Mayo Clinic, Phoenix, Arizona

**Keywords:** biceps femoris, snapping, lateral knee pain

## Abstract

This case involves a healthy male with painful lateral knee pain and snapping after a hyperextension injury. Initially, this was felt to be from a displaced lateral meniscus tear; however, he failed to improve after meniscal debridement. Further workup with an ultrasound and magnetic resonance imaging identified an aberrant biceps femoris anatomy. He was taken to the operating room and the aberrant slip was identified. A tenodesis of the aberrant slip to the biceps femoris was completed. This resolved the patient's pain and snapping, and he was able to return to all activities.

## Case Report

### History

**Fig. 1:**
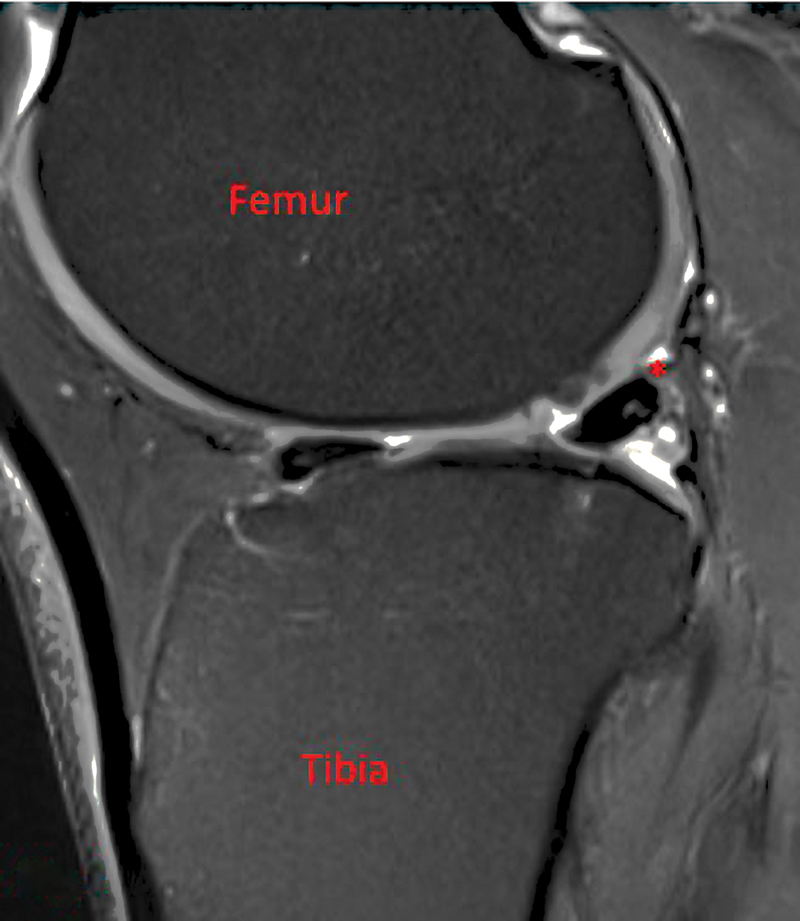
Magnetic resonance imaging showing lateral meniscus tear (*) and flipped fragment (◊).

**Fig. 2:**
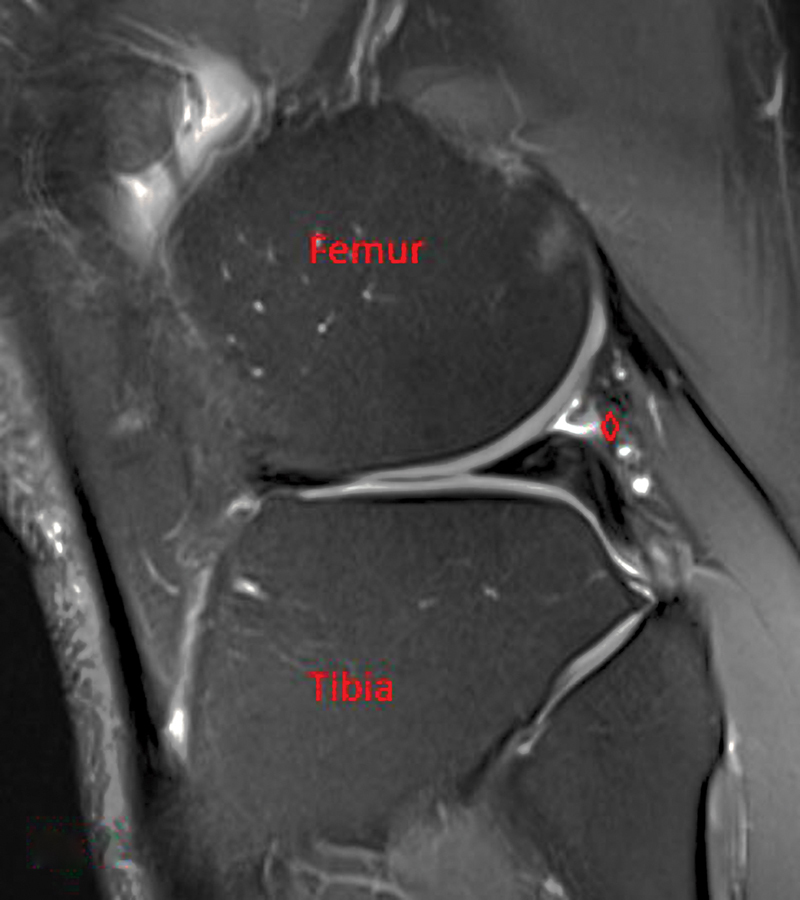
Magnetic resonance imaging showing lateral meniscus tear (*) and flipped fragment (◊).

A healthy 36-year-old man presented to the orthopaedic office via referral from a physical medicine and rehabilitation colleague for evaluation of right knee pain. He reported sustaining a hyperextension injury to the knee 2 weeks prior while in jiu-jitsu. He heard a pop but was able to continue training. That night his knee was painful and swollen. He was able to train regularly until sustaining a second injury a few weeks later. At this initial visit, he denied having knee pain with activities of daily living but was hesitant to squat or do any martial arts. Physical examination did not reveal any deformity, weakness, or instability. There was tenderness to palpation over the posterolateral knee. Snapping was not reproducible with flexion and extension maneuvers. The patient had a negative McMurray's test, and there was no joint line tenderness. The rest of the examination revealed good strength and neurovascular function. A magnetic resonance imaging (MRI) completed prior to evaluation was reviewed, demonstrating a lateral meniscus tear ( Fig. 1 ). This tear was felt to be flipped underneath the posterior horn with possible meniscocapsular separation ( Fig. 2 ). The patient elected to proceed with knee arthroscopy with meniscal debridement versus repair as indicated.

### Surgical Findings

The patient underwent a successful knee arthroscopy with partial lateral meniscectomy. Standard procedures for identification, preoperative antibiotics, and general anesthesia were followed. Intraoperative findings included a horizontal tear that had a displaced inferior leaflet just posterior to the joint space. It was pulled into the joint using a probe. It was felt that the tear was not repairable, and the meniscus was debrided using a combination of shaver and electrocautery. This was done until there was a stable meniscus. There was grade 2 chondral changes to the tibial plateau on the lateral side without loose chondral flaps. There were no significant changes to the lateral femoral condyle. The remainder of the arthroscopy was normal. The patient tolerated the surgery well and had a normal postoperative course. He was able to return to jiu-jitsu after 6 weeks of therapy.

### Clinical Outcome

**Fig. 3:**
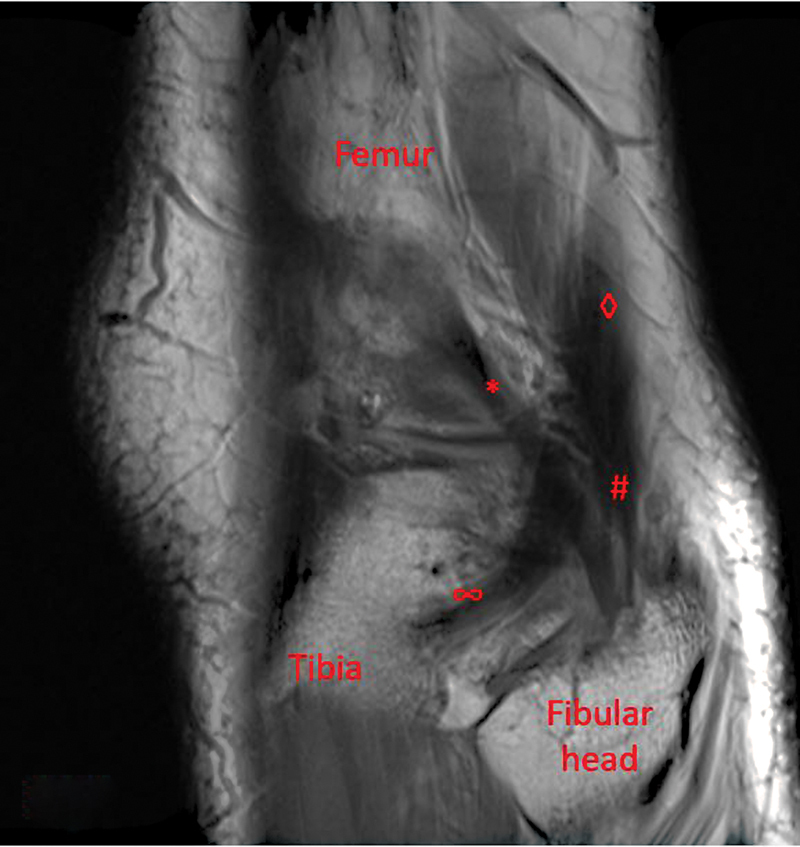
Magnetic resonance imaging showing lateral meniscus tear with flipped fragment (*), biceps femoris (◊), biceps femoris attachment into fibular head (#), and accessory attachment into tibia (∞).

The patient returned 5 months after surgery with a complaint of recurrent posterolateral knee pain and snapping. His symptoms were consistent with previous complaints and were significantly limiting his activities. On examination, the snapping was now reproducible in a figure-of-four positions with ranging the knee from flexion to extension. The remainder of this examination remained unchanged. Further evaluation with a dynamic ultrasound of the biceps femoris was completed to assess for a snapping biceps femoris tendon. The ultrasound showed a biceps femoris tibial attachment and was noted to be crossing the underlying thickened lateral collateral ligament. Dynamic examination with ultrasound was unable to reproduce the snapping experienced by the patient. Upon repeat review of the patient's previous MRI, an aberrant biceps femoris insertion was recognized ( Fig. 3 ). Given the correlation between examination and imaging studies, the patient was offered surgery to address the biceps femoris aberrant anatomy. The patient wished to proceed and was consented for biceps femoris slip reattachment to the posterior fibular head versus tenodesis to the biceps femoris.

### Surgical Technique

**Fig. 4:**
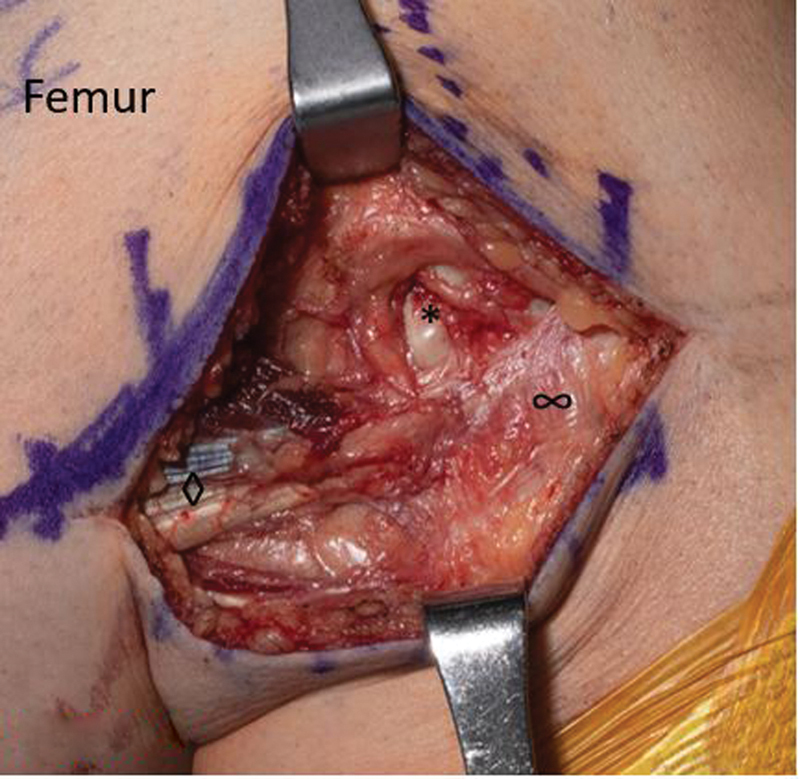
The anatomy prior to dissection. Intraoperative photos. Lateral collateral ligament (*), biceps femoris (◊), and accessory attachment into tibia (∞).

**Fig. 5:**
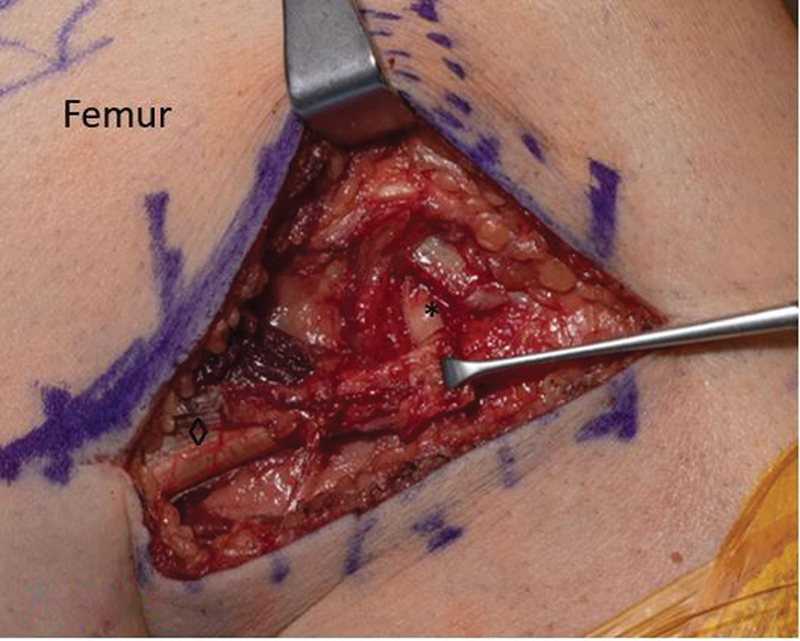
With accessory slip cut. Lateral collateral ligament (*) and biceps femoris (◊).

**Fig. 6:**
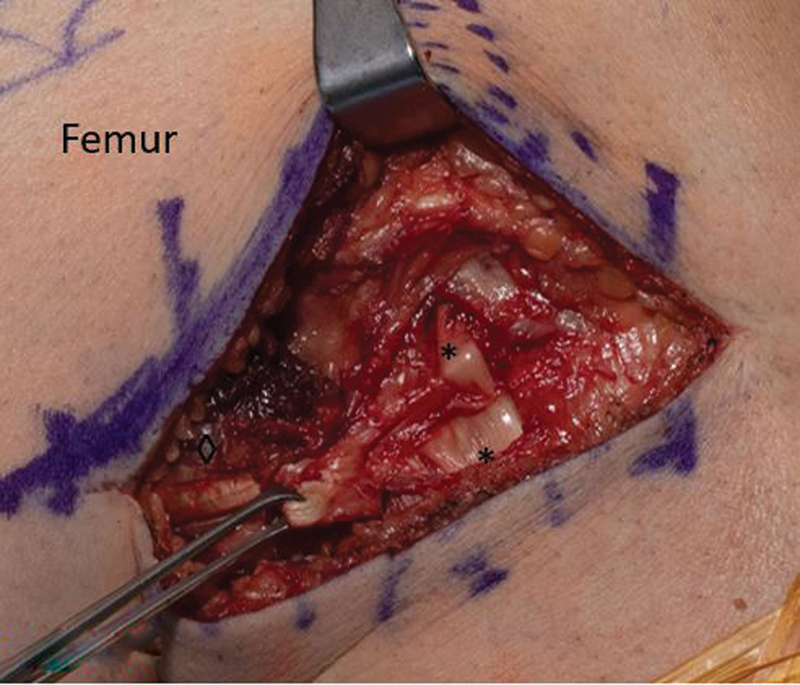
With the accessory slip retracted. Lateral collateral ligament (*) and biceps femoris (◊).

The patient was taken to the operating room following standard procedures for identification, preoperative antibiotics, and general anesthesia. A tourniquet was applied to the thigh, and the right lower extremity was prepped and draped in a sterile fashion. After time out, the extremity was exsanguinated, and tourniquet insufflated to 250 mm Hg. An incision was made along the biceps femoris, and superficial dissection was completed with electrocautery and Stevens' scissors. The biceps femoris muscle belly, tendon, and peroneal nerve were identified. Care was taken to protect the peroneal nerve throughout the procedure. Dissection of the biceps femoris was continued, and a slip was noted overlying the lateral collateral ligament with attachment to the tibia ( Fig. 4 ). This accessory slip was noted to be causing the patient's snapping and was released ( Fig. 5 ). After release, the biceps femoris was noted to still be under tension with the fibular slip remaining intact. The detached tibial slip was then tenodesed to the fibular attachment ( Fig. 6 ). No further accessory slips or aberrant attachments were noted under direct visualization through a full range of motion. No snapping was noted with flexion and extension maneuvers. The wound was irrigated and closed using 2–0 Vicryl, 3–0 Monocryl, and Prineo. He was placed in a long-hinged knee brace locked in extension with partial weight-bearing restrictions.

The patient was seen 2 weeks later for his first postoperative visit. He reported doing well but he felt a popping sensation to the knee while rolling over in bed a few days prior. He denied any other episodes of snapping and had no pain. No concerns were noted, and he was progressed to weight bearing as tolerated with the knee locked in extension. He was instructed to start home therapy for quad strengthening and was sent to physical therapy for passive flexion/active extension with progression to active flexion at 4 weeks postoperative. He continued to do well at his 6-week postoperative visit and was progressed to strengthening the hamstring. He reported no sensations of snapping to the knee. He was cleared to gradually progress back to jiu-jitsu over the following 6 to 12 weeks.

During physical therapy, at 10 weeks postoperatively, patient reported anterior knee pain with weight-bearing activities. Isometric testing on the ForceFrame (VALD Performance, Brisbane, Queensland) revealed a quadriceps limb symmetry index (LSI) of 58% at 60 days. Hamstring testing was not performed at this time secondary to pain. Squat assessment utilizing AMTI force plates and ForceDecks (VALD Performance) software was consistent with this, showing 19 and 14% asymmetry to the uninvolved side in eccentric mean force and concentric mean force, respectively. Balance assessment on the AMTI force plates was found to be unremarkable. He was given a home exercise program for quadriceps and hamstrings strengthening.

Upon returning for follow-up at the 12-week postoperative mark, he reported pain in the lateral hamstring muscle belly that did not radiate into the surgical site. At this time, trigger point dry needling (TDN) was performed to the biceps femoris in six locations along the muscle belly. Patient underwent 4 weekly sessions of TDN with complete resolution of symptoms. During this time, his home exercise program progressed to more intensive strength training.

At the 5-month postoperative mark, the patient reported no pain in the hamstring with activities of daily living and heavy strength training and no continued anterior knee pain. He underwent isokinetic strength testing on the Humac Norm (Computer Sports Medicine, Inc., Stoughton, MA) for quadriceps and hamstrings at 60/60 degrees/s. This revealed an LSI deficit of 20 and 18% in the involved quadriceps and hamstrings, respectively. He was again progressed to unilateral strength training with both closed and open kinetic chain exercises and given a return to running program.

At 7 months postoperatively, he denied any of the preoperative snapping. He was again tested for isokinetic strength. He improved significantly in both quadriceps and hamstring strength, with 30 and 8% increases, respectively, leading to an LSI of 3 and 14%. He reported no pain with all activities including running. At this point, he was medically cleared for all activities and return to jiu-jitsu with a graded return to sport plan.

## Discussion

The diagnosis of a snapping biceps is uncommon, and it is routinely mistaken for more common pathologies. 1 2 3 4 5 6 7 8 9 This case demonstrates an initially missed snapping biceps due to concurrent lateral meniscus tear after injury. Pain and snapping from the biceps femoris insertion can be due to anomalous anatomy of the insertion itself, dysmorphia of the fibular head or from trauma. 3 10 11 12 13 14 Saltzman et al also reported that the popliteus tendon, lateral collateral ligament, and iliotibial band can cause a popping sensation and snapping at the lateral knee. 15

The diagnosis is often made clinically, as the patient will typically have pain and snapping ranging the knee from terminal flexion into extension. Asking the patient to stand from a squat can confirm the diagnosis as the tendon can be visualized snapping over the fibular head. 13 Manual palpation during examination found to eliminate or reduce the patient's lateral popping and pain, points toward an extra-articular cause. 16 The provider should consider radiographs to assess the morphology of the fibular head. MRI can be used to evaluate abnormal soft tissue anatomy and rule out other causes of a painful lateral knee. Padovani et al also advocated for arthroscopy to definitively rule out intra-articular pathology. 17 Once diagnosed, conservative treatment is the first line of treatment and includes a period of activity modification, physical therapy, and anti-inflammatories. If the patient is unable to return to desired activities, surgical intervention to fix the anatomical abnormality can be offered.

**Fig. 7:**
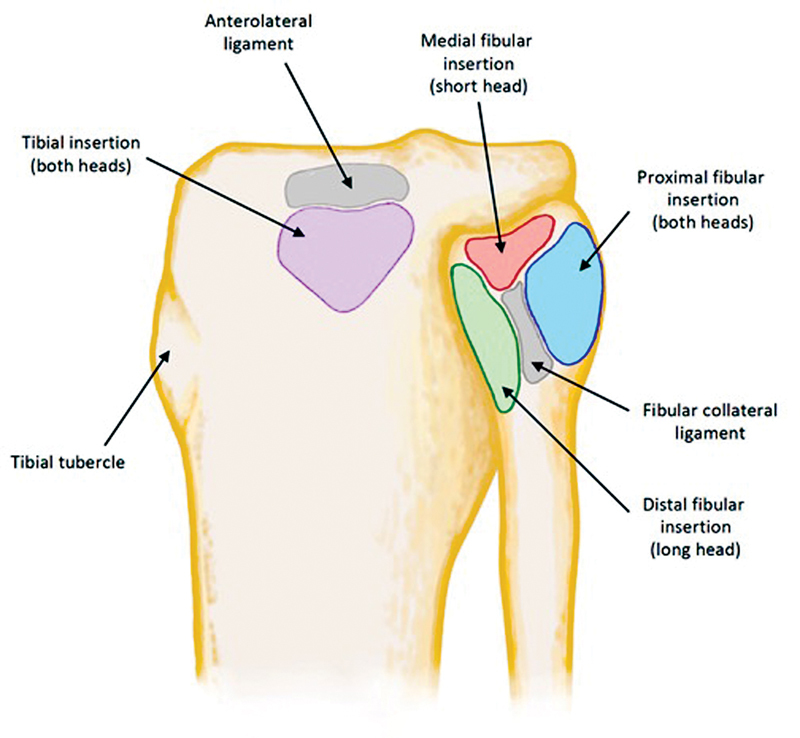
Anatomic footprints of biceps femoris, lateral collateral ligament, and anterolateral ligament.

The anatomy of the posterolateral knee is complex. Terry and LaPrade, 14 Branch and Anz, 18 and Tubbs et al 19 have completed quantitative analysis of the short and long heads of the biceps femoris insertions. Branch and Anz documented four reproducible anatomic footprints with dissection of 12 nonpaired cadaver specimens. These footprints were a proximal footprint of the biceps femoris (fibers from short and long heads) into the fibula, a distal lateral insertion upon the fibula mostly from the long head, a medial insertion to the fibula from the short head, and a tibial insertion with variable composition of the long and short heads ( Fig. 7 ). These four footprints were found to have consistent relationships to the lateral collateral ligament and anterolateral ligament.

To our knowledge, this is the second case described with aberrant biceps femoris anatomy treated with soft tissue tenodesis alone. Fritsch and Mhaskar 11 did a systematic review of 10 cases with only 1 being treated with a soft tissue tenodesis. All others were treated with some form of fibula revision, bone tunneling, tenodesis to bone, or anchors. In our case, we restored anatomy without a more invasive procedure. Despite only tenodesis of the aberrant slip, we were able to maintain tension of the native insertion and resolve the snapping. Postoperatively, the patient has done well and has returned to all preinjury activities.

## Conclusion

This case demonstrates the ease with which this diagnosis can be missed, and that when present, less invasive procedures can be done to correct aberrant anatomy.
